# The Lausanne Hospitality Model: a model integrating hospitality into supportive care

**DOI:** 10.1007/s00520-023-07726-2

**Published:** 2023-04-15

**Authors:** Kim Lê Van, Chantal Arditi, Lohyd Terrier, Françoise Ninane, Annie Savoie, Sylvie Rochat, Isabelle Peytremann-Bridevaux, Manuela Eicher, Béatrice Schaad

**Affiliations:** 1 Lausanne University Hospital CHUV, Lausanne, Switzerland; 2 Center for Primary Care and Public Health (Unisanté), Department of Epidemiology and Health Systems, University of Lausanne, Lausanne, Switzerland; 3 EHL Hospitality Business School, HES-SO, University of Applied Sciences and Arts Western Switzerland, Lausanne, Switzerland; 4 Patient Representative, Neuchâtel, Switzerland; 5 Institute of Higher Education and Research in Health Care - IUFRS, Faculty of Biology and Medicine, University of Lausanne, Lausanne, Switzerland; 6 Faculty of Biology and Medicine/Institute of Medical Humanities, University of Lausanne, Lausanne, Switzerland

**Keywords:** Hospitality, Hospital, Supportive care, Patient-reported experiences of care

## Abstract

**Purpose:**

Cancer care is undergoing a conceptual shift with the introduction of the principles of patient-centered care to support patients’ individual needs. These needs include those related to hospitality during cancer treatments. This paper aims to provide an extension of the supportive care framework by bringing in the hospitality approach inspired by the hotel industry.

**Method:**

The “Lausanne Hospitality Model,” integrating hospitality into supportive care, was developed through an iterative process, combining expertise in supportive care and health services research, communication, and the hotel industry.

**Results:**

This conceptual paper integrates hospitality and service sciences into the supportive care framework. The “Lausanne Hospitality Model” offers new insights into the notions of cancer journey, patient experience, services, and practices that may be involved when facilitating hospitality. While most concepts used in the model are based on prior research, they have not been combined previously. The model highlights the place of hospitality in the patient’s experience within cancer services and, by extension, its role in professional practice.

**Conclusion:**

Practices involved in the delivery of cancer care need to reinforce the importance attributed to hospitality services, as they impact patients’ experiences. By integrating the hospitality perspective into healthcare delivery and supportive care, this paper addresses previously theoretically overlooked aspects that impact patients’ experiences during cancer care.

## Introduction

Cancer prevalence and incidence have increased in most countries over the last decades. For many affected people living in countries with high Human Development Index, cancer has become a long-term condition due to more effective screening, diagnosis, and treatments, leading to increased survivorship. In Switzerland, more than 40,000 new cases of cancer are diagnosed each year [1], with estimates of growing prevalence in the next years and relative 10-year survival rates above 50% [2]. The periods of cancer diagnosis, treatments, and follow-up are often burdensome, in addition to the physical, emotional, social, functional, and financial consequences of cancer that affect patients’ quality of life [3, 4]. Thus, the question is not only whether patients survive a cancer diagnosis, but how (well) they survive.

Age, culture, economic status, profession, place of living, and family situation are only some of the dimensions affected by or influencing the subjective experience of people affected by cancer. This contributes to a growing recognition that offering standardized care to patients and their informal caregivers is not adapted to their individual supportive care needs. Since the late 1960s, there has been a conceptual shift in the administration of care that places a central focus on “understanding the patient as a unique human being” [5]. Since then, patient-centered care has encountered growing recognition as a fundamental predictor of healthcare quality and patient safety [6, 7]. Patient-centered care is defined as care that responds to patients’ physical, emotional, social, and cultural needs, where interactions with health professionals are compassionate and empowering, and where patients’ values and preferences are taken into account [8]. Patient-centered care is also one of the six core dimensions of quality of care according to the widely used framework developed by the *Organisation for Economic Co-operation and Development* (OECD) [9].

The shift to patient-centered care has led to the development of patient-reported experience measures (PREMs), which are measures of patients’ perception of their experience of care that can be used to evaluate the quality and patient-centeredness of care delivery. PREMs encompass the range of interactions that patients have with the health system relating to their (i) satisfaction (e.g., with information given by nurses and doctors); (ii) subjective experiences (e.g., staff helped with pain); (iii) objective experiences (e.g., waiting time before appointment); and (iv) observations of healthcare providers’ behavior (e.g., whether or not a patient was given discharge information). Various conceptual frameworks with dimensions of patient experiences have been developed to facilitate and standardize the use of PREMs [10, 11]. Most frameworks incorporate the following eight dimensions of patient-centered care [6]: (1) respect for patients’ values, preferences and needs; (2) information, communication, and education; (3) coordination of care; (4) physical comfort; (5) emotional support; (6) involvement of family and friends; (7) continuity and transition between healthcare settings; and (8) access to care. These measures are usually collected through patient surveys and collected data are used to identify areas with lower patient experience scores to inform quality improvement initiatives [12].

The recognition of the patient’s experience as a determining factor in their treatment is an essential point in supportive care. To achieve person-centered cancer care that goes beyond personalized treatment, a framework for targeted and tailored supportive care was developed from a task force created by the Ontario Cancer Treatment and Research Foundation [4], and has been adapted in many countries since then. The guideline defines supportive care in oncology as “the provision of the necessary services for those living with or affected by cancer to meet their physical, emotional, social, psychological, informational, spiritual and practical needs during the diagnostic, treatment, and follow-up phases, encompassing issues of survivorship, palliative care and bereavement” (p. 11).

In the present paper, we present an extension of the supportive care framework by drawing on contributions from the hotel industry and, more broadly, from the hospitality industry, in the way of conceiving the most appropriate services offered to patients in care settings. Brotherton [13] defines hospitality as “contemporaneous human exchange, which is voluntarily entered into, and designed to enhance the mutual well-being of the parties concerned through the provision of accommodation and food or drink” (p. 168). Grounded on the physical environment of a service organization, relationships with guests, or customer value creation, hospitality is recognized as a strong determinant of the value of a core service, supporting features and processes to improve service personnel’s and customers’ experience [14, 15].

The authors, a transdisciplinary team that included a person affected by cancer, critically examined the model of supportive care and reflected on the role of hospitality services in patient experience and supportive care. The team included two nurses, one physician, one patient expert, and five researchers with expertise in supportive care in oncology, health services research, communication, and the hotel industry. Together, they developed a model through an iterative process, turning to service science to identify levers of positive experience. Building on the synergies between the supportive care framework [4] and the hospitality perspectives coming from the hotel industry [16–18], the team developed “The Lausanne Hospitality Model: a model integrating hospitality into supportive care.”

**Fig. 1:**
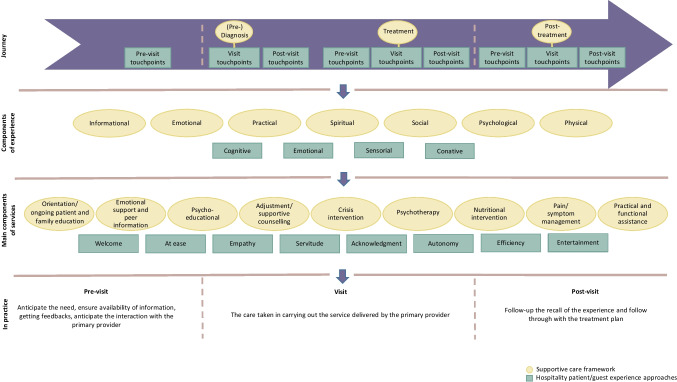
The Lausanne Hospitality Model: a model integrating hospitality into supportive care. Inspired by Fitch [4], Hunter-Jones et al. [17], Pijls et al. [18], Godovykh and Tasci [16]

The model is pictured in Fig. 1 and is organized in four sections: (i) the *Journey* section presents the key points of the patient/client trajectory; (ii) the *Components of experience* outline the dimensions considered in the patient/client experience in supportive care and hospitality; (iii) the *Components of services* present the dimensions defining service involved in supportive care and hospitality; and (iv) the last section, *In practice*, reviews the translation of the previous three sections into practical terms and managerial implications.

## The Lausanne Hospitality Model

### Cancer journey

The Supportive Care framework [4] organizes the cancer journey in terms of clinical steps: before the treatment (i.e., pre-diagnosis phase involving screening and assessment), during the treatment, and after the treatment during follow-up (i.e., post-treatment phase), which correspond to the traditional stages identified in the literature on the customer journey [19]. In the service industry, the customer journey refers to “a process or sequence that a customer goes through to access or use an offering of a company” (p. 336)” [20]. The customer journey analysis is based on identifying all the direct or indirect contacts—called touchpoints—the person has with the company and which participate in shaping the customer experience [21] (e.g., the call to the clinic to make an appointment, pre-visit confirmation, valet parking, post-visit feedback). Touchpoints occur at various points in time, across multiple channels, whenever a customer interacts with the company—product, service, brand, advertisement, and website [22, 23]. In customer experience management, not all touchpoints are equivalent and service interactions may encounter more expectations when the main offer is a service [19]. Indeed, from both a theoretical and a practical point of view, identifying key touchpoints will help designing a successful customer experience. Thus, according to Duncan and Moriarty [23], managing the customer journey and its different touchpoints will significantly impact the consumer’s relationship with the organization, the brand, or the service.

There are considerable differences between a patient journey within which free choice is not possible or limited by a disease, and a customer journey that is usually characterized by free choice. Nevertheless, adopting a customer experience approach in patient experience shows the advantage of bringing multiple touchpoints into the patient experience considerations, which introduces multiple stakeholders [17]. The hotel business is based on a process of continuous information gathering, carried out at each visit and each touchpoint, which allows to generate a personalized experience and adapts offers and services at each stay [24]. The aim is to improve, stay after stay, the quality of the service and the care of the customer/patient, to maintain consistency in all the stays, in terms of service and interaction with the institution. Thus, considering patient’s experience from the first touchpoint, even before the first visit at the clinic or hospital, makes it possible not only to pay attention to the quality of service since the very first contact, but also to identify patient’s needs to improve the care experience of the following steps. By gathering information about patients’ needs, preferences, and other relevant personalized dimensions, we can improve their care experience as their journey progresses, and ensure they feel a sense of continuity in their treatment.

### Components of experience

The model suggests here to extend *patient* experience to *human* experience, and consider the totality of environmental aspects that are actual levers to improve a hospital visit experience. Access to information and the way it is provided can be crucial for the person’s experience.

Facing cancer involves dealing with unexpected new life challenges and possibly unmet needs, impacting physical and psychological symptom burden in cancer patients [25]. Care responding to patient’s needs leads to better experiences of care for patients, which have been shown to be associated with higher levels of adherence to treatment processes, better clinical outcomes, better patient safety, and less healthcare utilization [26]. Based on the growing body of literature about patient experience, Wolf et al. [27] identified the following recurrent aspects within the various existing (and inconsistent) definitions of patient experience: “emotional and physical lived experiences, personal interactions, spanning across the [care] continuum, shaped by the organization/culture, and importance of partnership/patients involvement.” These aspects were integrated by the Beryl’s Institute in their definition of patient experience as “the sum of all interactions, shaped by an organization’s culture that influence patient perceptions, across the continuum of care” [28].

The supportive care framework [4] deconstructs the patient experience and targets the diverse needs that patients encounter during the cancer journey, contributing to the conceptual shift in which the patient is considered in his or her individuality. These needs may be informational (e.g., care processes, communication with caregivers, orientation, help with decision-making), emotional (e.g., fear, distress, anxiety, talking with peers, isolation), practical (e.g., transportation, child care, financial issues), spiritual (e.g., search for meaning), social (e.g., changes in roles, telling other people), psychological (e.g., anxiety disorders, self-image problems, sexual problems), and physical (e.g., pain, fatigue, weight changes). The intensity of these needs may also vary depending on the stage of the cancer journey. For instance, the need for information may be higher in the early course of illness, while the need for pain control may predominate as the cancer progresses.

From a service perspective in tourism industry, Godovykh and Tasci [16] define customer experience as “the totality of cognitive, affective, sensory, and conative responses, on a spectrum of negative to positive, evoked by all stimuli encountered in pre, during, and post phases of consumption affected by situational and brand-related factors filtered through personal differences of consumers, eventually resulting in differential outcomes related to consumers and brands” (p. 5). According to the authors, the *cognitive* component encompasses cognition, thoughts, educational and informative aspects, intellectual ability, rational capacity, knowledge, and memories. The *emotional* component—or *affective*—refers to affects, feelings, emotions, or mood experienced by a visitor. The *sensorial* component is described as sensations encountered by the customer. Finally, the *conative* components are related to behavior, involvement, act, and practice. If emotional and cognitive components of visitor experience overlap with patient’s needs, sensorial and conative components are supplementary and help understanding experience from a broader perspective, integrating the impact of the environment and professionals’ behavior, beyond the disease.

Visitor experience has been a key consideration for service organizations, since the economic development moved from a service economy to an experience economy [29]. When the priority shifted away from the employees’ behavior and the host’s viewpoint, considering guest or customer experience (e.g., interactions with staff, but also with the environment) became the main focus to effectively improve the hospitality of the organization [18]. Literature in marketing research and customer experience shows how the servicescape, namely the “built environment surrounding the service,” shapes customer expectations and influences the nature of customer experience [30]. Service setting influences the person’s interaction with the institution through various sensorial stimuli, serves as a facilitator by impacting the flow of activities, and conveys organizational culture by socializing both customers and employees [31]. In other words, the set of physical elements makes up the customer/patient experience. Consideration of hospitality-based experience requires then to view experience as a multidimensional construct, where the touchpoints directly influence sensory, affective, relational, or cognitive dimensions throughout pre-treatment, treatment, and post-treatment phases.

In light of the plural nature of the customer experience, researchers in hospitality management are multiplying the attempts of developing appropriate models showing what makes the experience and how it is organized. In the health field, the “Hospitality-oriented Patient Experience – HOPE” model developed by Hunter-Jones et al. [17] offers a framework for considering the patient experience with a hospitality orientation. At the junction of hospitality, health, and customer experience literature, the HOPE framework offers a hospitality-based approach to healthcare delivery and customer experience management, in which patients and hospital staff work together to improve the patient experience across every touchpoint in the care journey. By distancing itself from an outdated paternalistic model of care, the HOPE framework offers a vision of the patient, who goes from being a user to a shaper of services.

### Main components of services

The health literature emphasizes how targeted and tailored supportive care influences various dimensions of the healthcare pathway, for both patients and healthcare professionals involved. Indeed, the systematic review by Deneckere et al. [32] shows how the organization of treatments into care pathways reduces in-hospital complications and supports interprofessional teamwork, by influencing staff knowledge, interprofessional documentation, team communication, and team relationships. Coulter et al. [33] emphasize, for instance, that a better work environment, as well as patients who trust and respect their physicians, improve adherence, enhance self-care and result in higher ratings from patients. Bibby et al. [34], focusing on adolescents and young adults living with cancer, suggest a need for age-appropriate information and treatment facilities, access to emotional support services, contact with peers, and fertility information and services. The systematic review by Driessen et al. [35] highlights the added value of combining hospital formal care (e.g., provided by doctor, nurse, hospital psychosocial caregivers) with informal care (e.g., provided by volunteers, websites, online support programs, non-hospital therapy) in supporting both patients with cancer and their families in coping with the diagnosis: it has the potential to provide emotional and informational support, be cost-effective, and increase patient satisfaction with the care provided. Additionally, a strong need for emotional support was also identified as a main psychosocial issue when people were told their cancer was incurable [36]. Finally, the literature synthesis of Kandampully et al. [14] indicates that although limited, literature on customer experience management shows that factors such as aesthetics, ambiance, lighting, social, services design, emotions, and customer-customer interactions have a significant impact on the customer experience in the hospitality industry.

Targeting patient’s needs when facing cancer allows for the development and delivery of relevant services within the health institution. The framework of supportive care is supplemented by recommendations for practice to be used on a managerial level. Thus, several types of services are highlighted as having a benefit for cancer patients and their family members: facilitating orientation and introducing the oncological health system, providing emotional support, providing an opportunity to learn about and develop coping skills, offering regular assistance, setting up a crisis intervention program, carrying out psychotherapy sessions if necessary, giving advice regarding nutrition, reducing distress due to symptoms, and designing services to assist practical and functional matters [4]. As specified by the authors, the challenge lies in ensuring that information is provided to patients and their relatives and that access to services is facilitated.

The services mentioned in the framework of supportive care have the advantage of taking into account clinical, practical, and environmental dimensions that a health institution should address. In order to develop an instrument for measuring hospitality, Pijls et al.’s [18] qualitative analysis of interviews with hospitality experts and customers of service organizations resulted in the subdivision of hospitality into nine experiential dimensions. The *Welcome* dimension refers to a warm reception and atmosphere. Feeling *at ease* describes feeling at home, confident, safe, and relaxed. The dimension of *Empathy* relates to the idea that the organization understands what guests want and need. *Servitude* represents the feeling that the organization genuinely wants to serve the guest. The *Acknowledgment* dimension involves the feeling of being taken seriously and experience contact. *Autonomy* refers to the level of control that a guest has over what happens. *Efficiency* is associated with smooth procedures and the ease of arranging what guest wants. Finally, the *Entertainment* dimension refers to the ability of the organization to provide options for pastime (e.g., magazine, drinks).

While the healthcare approach bases its services on clinical dimensions, the hospitality approach offers a deconstruction of the service around the factors influencing the reception of these services by any consumer. The feeling of being welcomed, comfortable, heard, recognized, and entertained, with easy access to different services, but also in possession of the resources to act autonomously, are all factors that will shape the patient/client's evaluation of the service and, by extension, the quality of their experience. Thinking about services offered to cancer patients through a hospitality lens implies revisiting the very definition of what a hospital service entails.

### In practice

The fourth and final section of the model highlights the behavior and capabilities of the organization to execute service in practice, as a determining factor of the guest/patient’s experience of hospitality. In the last two decades, hospitality has taken a key role in the health sector and several hospitals around the globe have taken inspiration from the hotel and guest services industry to redesign their environments accordingly. For instance, the Texas Children’s Hospital of Houston (USA) and the Disney Institute collaborated in the development of a tailored immersive experience program to enhance the patient and family journey [34]. A similar approach has been adopted at Christ Hospital Health Network, in Cincinnati (USA), with the help of the Ritz-Carlton. The Mayo Clinic in Rochester (USA) built their worldwide reputation around the priority that “the needs of the patient come first:” in addition to a smartphone application and a concierge service, the clinic places great importance on the interpersonal skills of their staff members during the recruitment processes [37, 38]. The Centre Hospitalier Universitaire de Montréal CHUM (Canada), as well as every public hospitals in Paris (France), have adopted an SMS information system to guide the patient and inform family members about treatment follow-ups. Montefiore Hospital in New York (USA) have created a patient and customer service department to integrate hospitality features into healthcare [39]. The Henry Ford West Bloomfield Hospital, outside Detroit (USA), offers a uniformed valet service, patient meals served on demand 24 h a day, and in-room massages [40]. Finally, The Farrer Park Company in Singapore offers an integrated healthcare and hospitality complex [41]. These examples are just some of the initiatives showing how the health sector has been inspired by the hotel industry to consider services in patient care.

In their “Hospitality in patient experience framework,” Hunter-Jones et al. [17] outline recommended practices for providing hospitable services, organized in pre-/during/post-visit. Before the visit, services giving access to the information have to be simple, clear, and at ease (e.g., knowing and understand the goal of the appointment, how to access the meeting point, identifying who can be contacted in case of need). During the visit, the process needs to be smooth and swift, with a direct understanding of who the interlocutors are, what is discussed or treated and what the next steps are (e.g., available interlocutors, identification of the priority contact persons, awareness, and instructions on post-visit activities). Finally, the post-visit stage also deserves care, by ensuring a correct follow-up of the experience (e.g., post-visit feedback, easy access to information provided by healthcare professionals).

Working to co-creating values with customers, investigating in quality management or focusing on customer orientation are all strategies working towards a shift from service *logic* towards service *orientation* [42, 43]. In other words, hospitable service offerings and high-quality performance provided by employees are at the core of the emotional experience that impacts long-term customer satisfaction [44].

## Conclusion

The aim of this paper was to broaden the understanding of supportive care, by integrating hospitality into supportive care and offering an extension to the framework developed by Fitch [4]. The “Lausanne Hospitality Model: a model integrating hospitality into supportive care” considers components and perspectives that are usually treated independently in the literature. Integrating hospitality components into supportive care is based on the argument that expanding the range of services provided in the care journey can enhance the patient’s overall experience.

The commitment to delivering high-quality informational, emotional, and practical services transcends geographical boundaries. Nonetheless, the development of this model was tailored for implementation within a particular socio-cultural environment. Before applying the model in a different country, it should be adapted to fit its cultural, socio-economic, and political environment. The definition of what constitutes a quality service, what shapes the patient experience, the level of sensitivity to the physical environment, and how to translate these services into practice would require adjustments accordingly.

The model positions itself as part of the innovating analytic turn that aims at considering patient experience in its totality and complexity, considering not only the clinical characteristics of the individual, but also the extent to which environmental, organizational, emotional factors may determine his or her experience. This paper addresses previously overlooked aspects of patients’ hospital experiences by integrating a hospitality perspective into healthcare delivery and supportive care in the hospital.
